# Case of Paralysis

**Published:** 1807-05

**Authors:** 


### *To the Editors of the Medical and Physical Journal.*

GENTLEMEN,

AS the virtues of many of our most valuable remedies have been unexpectedly discovered through accident, I have taken the liberty of sending you the following case, which I hope will not prove altogether unserviceable.

Mrs. O. aged upwards of 60, who had been for many years subject to a slight paralytic affection, was in October last, suddenly seized with an abolition of nearly all sense and voluntary motion, paralysis of the right side of the body, stertorous breathing, and a great disposition to sleep, with a slow and full pulse.—A blister was applied to the back of the neck, and a purgative draught with great difficulty administered; she continued thus three or four days, and notwithstanding the application of leeches, and exhibition of those remedies, as are in such cases usually resorted to, she made but little or no progress towards recovery.

 Having

Having ordered her a mixture of T. Valer. vol. and an embrocation as follows: ℞. Aq. ammon. pur. ol. olivar. aa. ℥vi. ol. succini ℥i. m. ft. embrocat. with which to bathe the side affected; her attendants, by an unaccountable mistake, used the mixture for the embrocation, and actually gave her two tea-spoonfuls of the latter, every three or four hours, so that by the next visit she had taken nearly the whole. I was not a little surprised to find my patient much better, in every respect: she had recovered some motion in her leg and arm, her speech was uncommonly improved, which on the day before, was unintelligible; finding her going on thus well, I directed her to continue taking the embrocation, which she did, and mended so rapidly, in a short time she required no further attendance, and now enjoys as good a state of health as prior to the attack; and should she, (as is most likely) have another fit, I shall instantly have recourse to the same medicine, and hope with the same success.

TYRO.

*February* 4, 1807.

